# Machine learning-enabled estimation of cardiac output from peripheral waveforms is independent of blood pressure measurement location in an in silico population

**DOI:** 10.1038/s41598-025-10492-2

**Published:** 2025-07-15

**Authors:** Lydia Aslanidou, Georgios Rovas, Ramin Mohammadi, Sokratis Anagnostopoulos, Cemre Çelikbudak Orhon, Nikolaos Stergiopulos

**Affiliations:** Laboratory of Hemodynamics and Cardiovascular Technology (LHTC), EPFL, Lausanne, Switzerland

**Keywords:** Non-invasive monitoring, Neural networks, Cardiac output, Applanation tonometry, Machine learning, Pressure waveforms, Biomedical engineering, Cardiovascular diseases, Physiology, Anatomy, Biomarkers, Health care

## Abstract

Monitoring of cardiac output (CO) is a mainstay of hemodynamic management in the acutely or critically ill patient. Invasive determination of CO using thermodilution, albeit regarded as the gold standard, is cumbersome and bears risks inherent to catheterization. In the pursuit of noninvasive methods, prediction of CO from arterial pressure waveforms (even uncalibrated) through AI has garnered increased attention. In the current study we investigate the effect of peripheral blood pressure measurements’ (i) location, (ii) calibration and (iii) injected additive noise on the performance of AI-based CO estimation. A large previously generated virtual cohort of n = 3818 subjects with varied hemodynamic profiles served as data bank for arterial pulse waves and reference CO values. Two-layered convolutional neural networks (CNN) yielded CO estimates based on entire pressure traces from the radial, superficial temporal and common carotid arteries. The predictive ability of the CNN models was comparable across arterial locations (r ≥ 0.97, nRMSE  ≤ 4.4%) and only slightly compromised following pressure signal normalization from 0 to 1 (r  ≥ 0.93, nRMSE ≤ 7.8%). Following noise injection, model performance was preserved (r  ≥ 0.94, nRMSE ≤ 6.6%) yet became more sensitive to loss of calibration (r ≥ 0.78, nRMSE ≤ 12.2%). Our study highlights the interchangeability of potential blood pressure measurement locations in the quest for pressure-based CO estimation.

**Supplementary Information:**

The online version contains supplementary material available at 10.1038/s41598-025-10492-2.

## Introduction

Cardiac output (CO), defined as the volume of blood pumped by the heart into the systemic circulation in liters per minute, is a central component of effective hemodynamic management in perioperative and intensive care. At present, highly invasive (transpulmonary or pulmonary artery) thermodilution remains the clinical gold standard for CO monitoring. Yet thermodilution-based determination of CO is cumbersome, impractical and bears risks inherent to catheterization. A plethora of minimally invasive and noninvasive CO monitoring techniques have been proposed in recent years, yet their implementation in clinical practice is hampered by accuracy and precision concerns^1^. Benchmarking against the reference thermodilution method is further complicated by the lack of universally accepted and rigorous assessment criteria^2,3^. There is a pressing need for simpler, more reliable and performant noninvasive methods of measuring CO to improve patient outcomes and optimize therapeutic guidance.

At varying levels of invasiveness, techniques for CO monitoring in clinical practice are burdened with different limitations. Highly invasive methods, including pulmonary artery or transpulmonary thermodilution, pose technical challenges, use up resources and have mixed outcomes on mortality^4^. Minimally invasive ones, such as esophageal Doppler or pulse contour methods, besides still requiring calibration through catheterization in some cases, inherently lack reliability for certain clinical scenarios of fast changes in vasomotor tone, such as septic shock^5,6^. Noninvasive CO monitoring techniques, while circumventing the need for patient catheterization, have yet to gain a foothold in standard clinical practice: none of the existing noninvasive methods has heretofore been deemed interchangeable with the reference thermodilution method^7^.

Noninvasive CO monitoring technologies currently employed in clinical practice include two-dimensional (2D) and three-dimensional (3D) transthoracic echocardiography (TTE) as well as cardiac magnetic resonance imaging (CMR). TTE enables real-time, bedside evaluation of cardiac function and hemodynamics, using Doppler and volumetric methods to estimate stroke volume and CO^8^. While 2D TTE is widely adopted due to its accessibility and cost-effectiveness, its interchangeability with thermodilution has come under scrutiny^9^. 3D TTE offers enhanced accuracy by minimizing geometric assumptions, thus improving the precision of CO estimation^8^. Despite its advantages, TTE-based cardiac output estimation is limited by factors such as poor acoustic windows in patients with emphysema and technical challenges in those undergoing mechanical ventilation or recovering from cardiothoracic surgery^10,11^. CMR, considered the noninvasive reference standard for volumetric and functional cardiac assessment, provides CO measurements via phase-contrast velocity mapping^12^. However, its clinical application is limited by high cost, lengthy acquisition times, and the need for patient transport to MRI-compatible facilities, reducing its feasibility in intensive care settings. Both of these noninvasive modalities are inherently intermittent, operator-dependent in the case of echocardiography, and unsuitable for continuous hemodynamic monitoring in critically ill patients. As an alternative, fully noninvasive arterial pressure–based cardiac output technologies are gaining traction.

The advent of AI-based methods has revolutionized wearable technology and brought about an increase in noninvasive estimation potential. Machine learning algorithms can demonstrably extract the wealth of hemodynamic information embedded in a single blood pressure waveform, notably for pulse wave velocity estimation from in vivo carotid^13^, radial^14^, or combination of carotid, radial and brachial waveforms^15^. Fourier-based machine learning has recently been applied to reconstruct central pressure waveforms from in vivo brachial cuff measurements^16^. These applications underscore the growing potential of machine learning to advance cardiovascular diagnostics by extracting clinically relevant information from routinely acquired physiological signals. In a prior study, radial artery pressure waveforms fed to an artificial neural network were found to yield reasonable estimates of CO in a virtual population, even when treated as uncalibrated pressure traces^17^. It thus appears that the calibrated, and in fact even the raw uncalibrated, arterial pressure signal at the radial site contains sufficient information to accurately estimate CO, based on in silico data.

We hereby investigate additional peripheral arterial sites, each characterized by different topology and proximity to the heart, to assess whether local calibrated and uncalibrated pressure traces could be used to derive CO as accurately as the ones obtained at the radial artery. The study aims to identify the optimal arterial site for waveform measurement that yields the greatest accuracy in model-based estimation of CO. It implicitly addresses a fundamental physiological question: As the arterial pulse wave propagates through the vascular tree—encountering numerous bifurcations along different arterial paths—is the information encoded within the arterial pressure waveform preserved? Importantly, does the net distance from the heart play a role in the accuracy of our estimations? We assess the AI-based estimations of CO from the entire pressure waveform at three arterial locations and the resulting models’ resilience to pressure signal normalization and noise addition.

## Methods and materials

### Arterial sites of interest

Following the vascular tree’s hierarchical branching structure, each peripheral location is reached from the heart through a unique arterial path. The sum of bifurcations encountered along the transit path reflects the arterial location’s unique topological complexity, in a variant of the Weibel generation number^18^.

We select three palpable peripheral arteries, namely the left common carotid, left superficial temporal and left radial arteries, by virtue of ease and accessibility of local tonometric measurements. Applanation tonometry is a noninvasive method for acquiring arterial pressure waveforms that is used extensively in vascular research (see^19^ for a detailed description). Each location’s distinct topology, in terms of branching junctions and distance from the heart, is presented in Table 1.

**Table 1:** Representative topological characteristics of the three arterial locations of interest (based on the reference arterial geometry and arborescence in Reymond et al.^20^).

Arterial site	# Bifurcations encountered	Typical proximal distance (cm)
Left common carotid	2	20
Left superficial temporal	6	46
Left radial	4	79

### In silico population

A large in silico population of n = 3818 synthetic hemodynamic cases has been previously generated and used in a range of hypothesis-driven and proof-of-concept studies for non-invasive hemodynamic monitoring^17,21,22^. The virtual database was populated by means of a validated 1D model of the systemic circulation^20,23^, via sampling of demographic and anthropometric parameters within physiological value ranges. The original publication contains a detailed description of the virtual cohort’s generation and cardiovascular metrics^24^. Metrics pertinent to the current work are reported below.

**Fig. 1:**
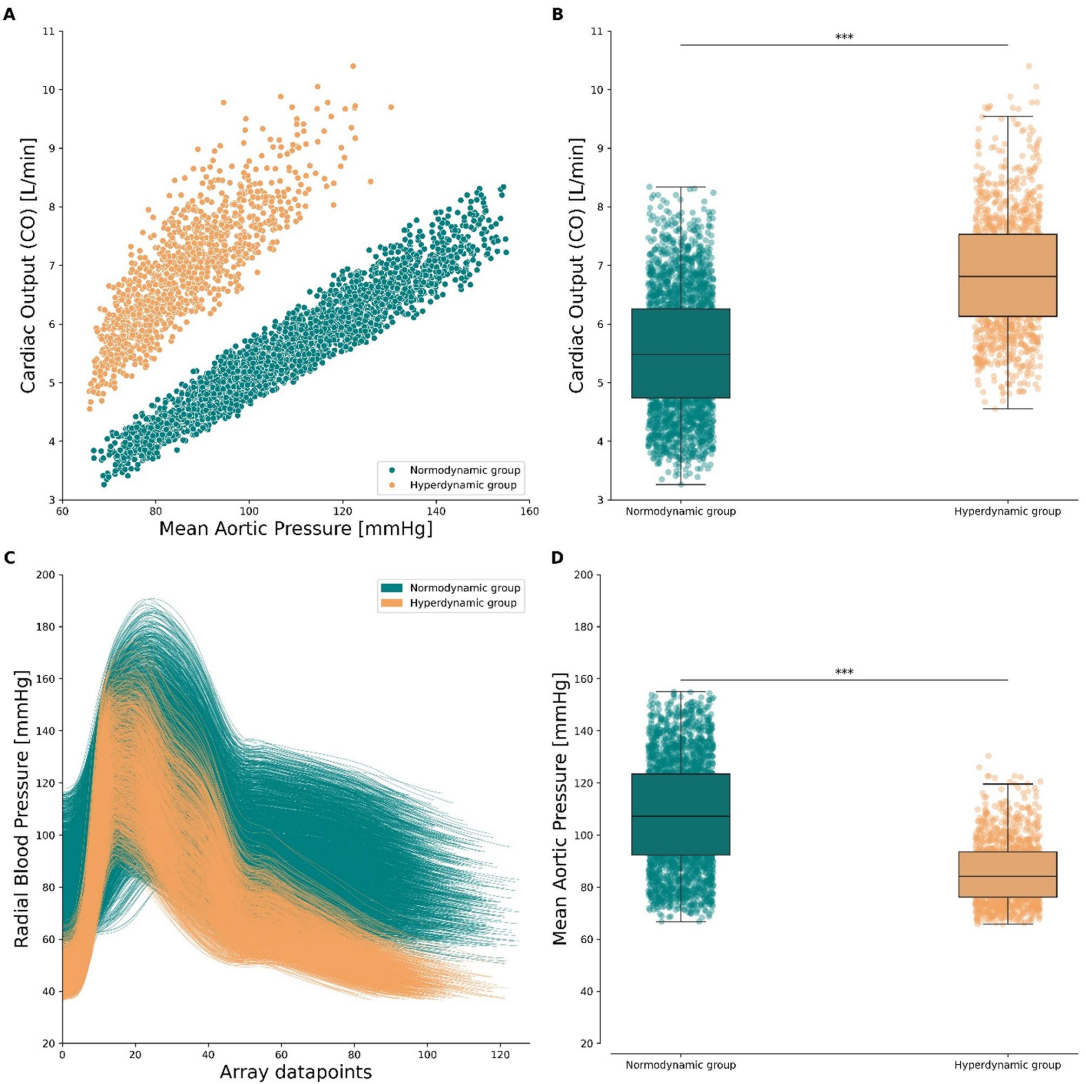
Subpopulations within the in silico dataset. (**A**) Cardiac output against mean aortic blood pressure for all n = 3818 synthetic cases. The two clusters are grouped using a Gaussian Mixture Model in a normodynamic (teal color, n = 2620) and a hyperdynamic (orange color, n = 1198) group, based on cardiac output levels. (**B**) Cardiac output values in the normodynamic and hyperdynamic group. Mean ± SD is (5.5 ± 1.0 L/min) versus (6.9 ± 1.0 L/min) respectively. (**C**) Raw radial pressure traces in the two groups. (**D**) Mean aortic pressure in the normodynamic and hyperdynamic group. Mean ± SD is (108.1 ± 20.2 mmHg) versus (85.8 ± 11.8 mmHg) respectively. Boxplots represent the interquartile range (25th to 75th percentile), central line indicates the median. ***p < 0.001 (non-parametric Mann–Whitney U test).

The in silico dataset comprises two distinct subpopulations that were identified post hoc based on cardiac output values: a normodynamic group with normal CO (5.5 ± 1.0  L/min, n = 2620) and a hyperdynamic group with significantly elevated CO (6.9 ± 1.0 L/min, n = 1198) as illustrated in Fig. 1A,B. This data-driven grouping was not predefined but emerged from analysis of the CO distribution (Fig. 1A). Radial pressure wave morphology differs between the two groups (Fig. 1C), with additional individual examples shown in Supplementary Material Fig. S1. The mean aortic pressure is significantly lower in the hyperdynamic group (85.8 ± 11.8 mmHg) than in the normodynamic group (108.1± 20.2 mmHg; Figure 1D). The heart rate does not differ substantially between groups: the hyperdynamic group shows a mean HR of 83.9 ±  7.7 bpm (min-max range of [62.5, 101.6]), while the normodynamic group has a mean HR of 81.9 ± 8.3 bpm (min–max range of [61.1, 101.6]). Overall, the hyperdynamic group has higher CO, lower systemic vascular resistance, lower mean aortic pressure and similar HR as the normodynamic group—a pattern that would suggest a vasodilated high-output state.

A summary of the blood pressure data at the ascending aorta as well as at the three peripheral sites under consideration (left superficial temporal, left common carotid and left radial artery) for both subpopulations can be found in Table 2.

**Table 2:** Descriptive value ranges for blood pressure in silico population (n = 3818) at arterial locations under consideration.

Arterial site	Mean BP	Systolic BP	Diastolic BP	Pulse pressure
	(min, max), *µ* ± *σ*	(min, max), *µ* ± *σ*	(min, max), *µ* ± *σ*	(min, max), *µ* ± *σ*
Normodynamic group				
Ascending aorta	[67, 155], 108 ± 20	[77, 187], 125 ± 25	[57, 128], 91 ± 16	[11, 81], 33 ± 12
Left common carotid	[67, 155], 108 ± 20	[81, 188], 127 ± 24	[56, 128], 91 ± 15	[13, 84], 36 ± 12
Left superficial temporal	[63, 147], 102 ± 19	[83, 179], 123 ± 22	[53, 121], 86 ± 14	[18, 82], 37 ± 10
Left radial	[61, 144], 100 ± 19	[82, 191], 129 ± 24	[51, 118], 83 ± 14	[20, 99], 46 ± 14
Hyperdynamic group				
Ascending aorta	[66, 131], 86 ± 12	[79, 181], 118 ± 20	[43, 78], 55 ± 7	[27, 107], 63 ± 18
Left common carotid	[66, 131], 86 ± 12	[81, 182], 119 ± 20	[42, 78], 55 ± 7	[29, 108], 64 ± 17
Left superficial temporal	[61, 120], 79 ± 11	[79, 168], 112 ± 17	[38, 70], 49 ± 6	[32, 101], 63 ± 15
Left radial	[59, 116], 76 ± 11	[88, 177], 127 ± 17	[36, 67], 47 ± 6	[42, 118], 80 ± 16

Central ascending aortic values provided for reference. *BP* blood pressure, *µ*: mean, *σ*: standard deviation. Values are in mmHg.

### Pressure waveform preprocessing

In the current work, we use deep learning models to predict cardiac output based on full-cycle blood pressure waveforms. In our virtual population, heart rates vary (with mean ± SD 83 ± 8 bpm) resulting in pressure waveforms of different lengths. To standardize inputs for the model while preserving waveform morphology, we set a fixed single-cycle waveform vector size of 150 datapoints. Hence all pressure waveforms are either down- or over-sampled to uniformize their array length. Resampling to the discretization space of R^150^ is performed with MATLAB’s *interp1* function. Although sampling rate discrepancies between clinical measurement devices (e.g., 128 Hz for SphygmoCor CP, 1000 Hz for PulsePen) are not present in our synthetic dataset, the resampling strategy adopted here would also be applicable in real-world settings to harmonize signals acquired at different temporal resolutions. We perform amplitude normalization to obtain the uncalibrated (normalized) signals by dividing each waveform by its range over the entire cardiac cycle (i.e., P − P_min_/P_max_ − P_min_).

**Fig. 2:**
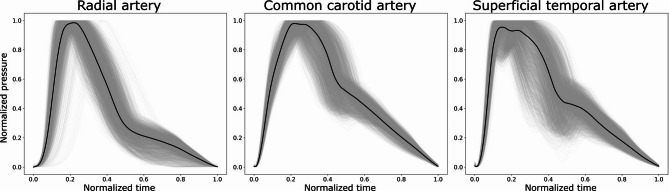
Uncalibrated (normalized) pressure waveforms of the entire virtual population (n = 3818) at the arterial sites under consideration (considered at the left anatomical side). Median trace is highlighted in black. Each heart cycle is rescaled here to span 0 to 1 for shape comparison.

Distinct morphologies of pressure waveforms at the three arterial locations are illustrated in Fig. 2.

### CNN model architecture

#### Hyperparameter tuning

**Fig. 3:**
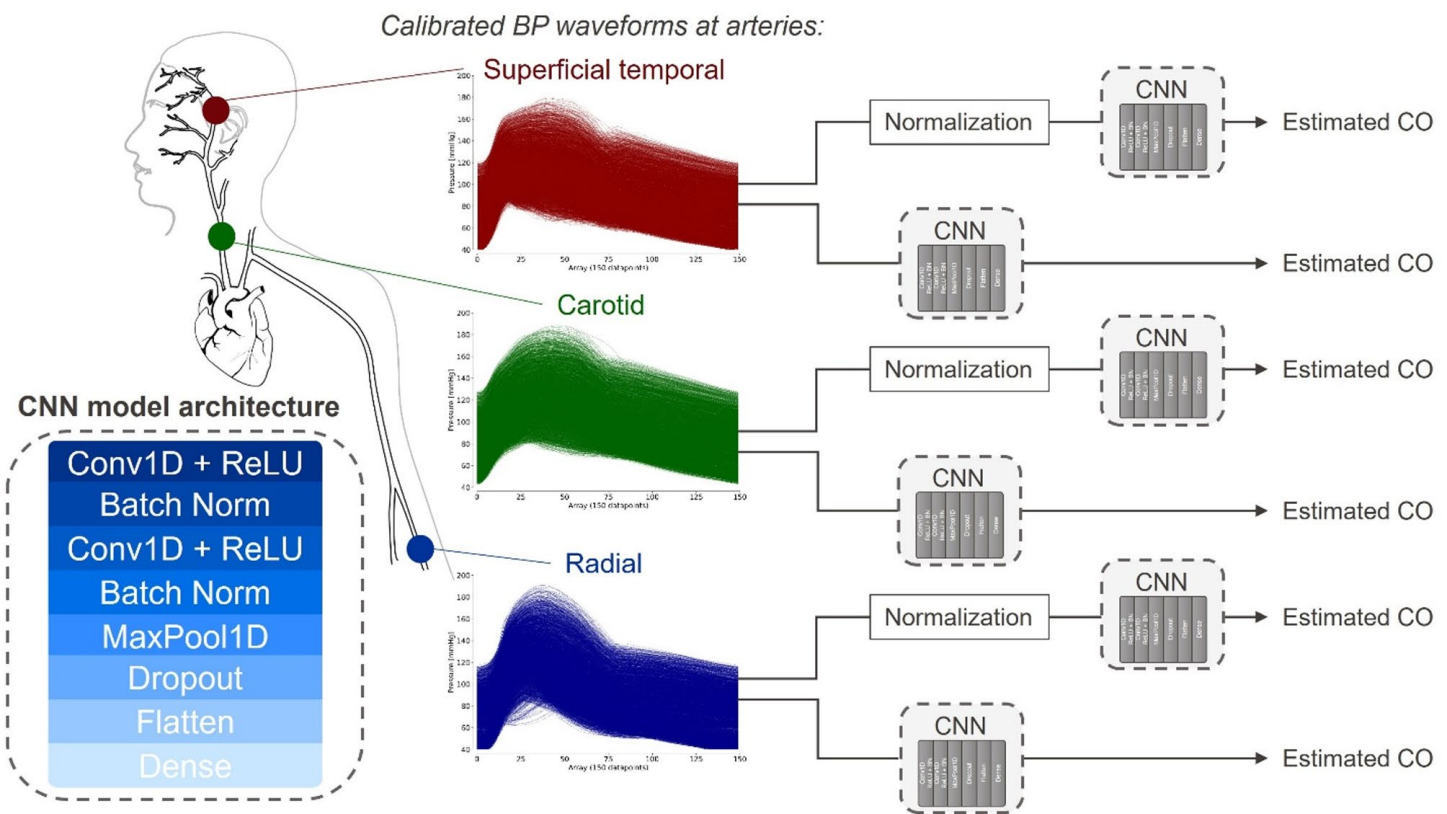
Six CNNs, whose hyperparameters are obtained through Bayesian optimization with stratified fivefold cross-validation, are trained on calibrated and uncalibrated (amplitude-normalized from 0 to 1) pressure traces obtained at three tonometry-ready arterial locations. Bottom left: panel depicting the CNN architecture.

A 1D Convolutional Neural Network (CNN) receives as input either the calibrated or the uncalibrated (amplitude- normalized) blood pressure waveform from each of the 3 arterial locations to estimate the cardiac output value, thus resulting in 6 different CNNs (Fig. 3). The input for each location-specific CNN is the set of pressure waveforms of the specific location, where each waveform is an array of 150 points, obtained as described above. The sequential CNN model is constructed with two 1D convolutional layers, each followed by batch normalization to stabilize training. A 1D max pooling layer is then applied for downsampling, followed by a dropout layer to reduce overfitting. The output is flattened and a dense (fully connected) layer is used for regression. For each of the 6 models, Bayesian optimization is employed to define the learning rate, batch size, number of filters, kernel size, dropout rate and the number of units in the fully connected (dense) layer. For each set of hyperparameters during Bayesian optimization, the model is evaluated through five-fold cross validation (using *StratifiedKFold* from *sklearn*), i.e. the dataset used for hyperparameter tuning is split into 5 subsets and the model is evaluated on different combinations of training and validation sets, ensuring that every datapoint is used for validation exactly once. The optimal number of epochs is determined using the early stopping callback function to avoid overfitting. All hyperparameters are summarized in Table S1.

#### Workflow

Following the methodology proposed by Bikia et al.^17^, the full dataset of 3,818 samples was split into training, validation, and test sets in a 60%/20%/20% ratio (2290/764/764 cases, respectively). To ensure the generalizability of the trained models, both the normodynamic and hyperdynamic groups (identified in the “In silico population” Section) are adequately represented across the training, validation and test sets through stratified sampling (through the argument *stratify* of sklearn’s *’train test split’* function). Furthermore, to ensure a fair comparison of model performances, each of the six models undergoes hyperparameter tuning separately. It is noted that the holdout (test) set remained unseen during model training and hyperparameter tuning. It was reserved exclusively for the final CNN performance evaluation to provide an unbiased assessment of model performance on unseen data.

### Noise injection

Synthetic waveforms, while useful for proof-of-concept or methodology development studies, represent idealized noise-free records far from real-world blood pressure acquisitions. Applanation tonometry at the radial artery and other locations is fraught with motion and interference artefacts resulting in baseline wandering, low- and high-frequency noise and amplitude spikes. Introducing noise to the synthetic pulse waves mimics some of the frequently observed measurement artefacts, allowing us to assess noise resilience of the CNN models, in an approach similar to other in silico based studies^14,17,25,26^.

Given the abolition of frequency information at the preprocessing step of the current framework (“Pressure waveform preprocessing” Section), we consider two sources of noise: (i) Gaussian noise, and (ii) discrete random perturbations in the form of spikes. For each data point in the pressure waveform vector *x* = [*x*_1_,*x*_2_,…,*x*_150_] Gaussian noise *n*_*i*_ ∼ *N* (*µ,σ*^2^) is added. We consider (*µ* = 0*.*7, *σ* = 1*.*0) for the calibrated and (*µ* = 0, *σ* = 0*.*1) for the uncalibrated signals. The amplitude of each spike is set to 10% of the maximum value of the signal, i.e. *A* = 0*.*1·*max*(*x*). To allow spikes to be either positive or negative, their amplitude is further scaled by a random factor drawn from a uniform distribution *U*(− 1,1), resulting in final spike amplitudes A′ ∼ *U*(− A,A). Spikes are added at randomly selected indices *I* comprising 20% of the 150 waveform points.

Concisely:

$$ \begin{gathered} \tilde{x}_{i} = x_{i} + n_{i} + r_{i}\, {\text{for}}\, i \in I \;{\text{where}}\, x_{i} \sim {\mathcal{N}}\left( {\mu ,\sigma^{2} } \right)\, {\text{and}}\, r_{i} \sim A^{\prime } \hfill \\ \tilde{x}_{i} = x_{i} + n_{i}\, {\text{for}}\,\,\,\,\,\,\,\,i \notin I \hfill \\\end{gathered} $$

**Fig. 4:**
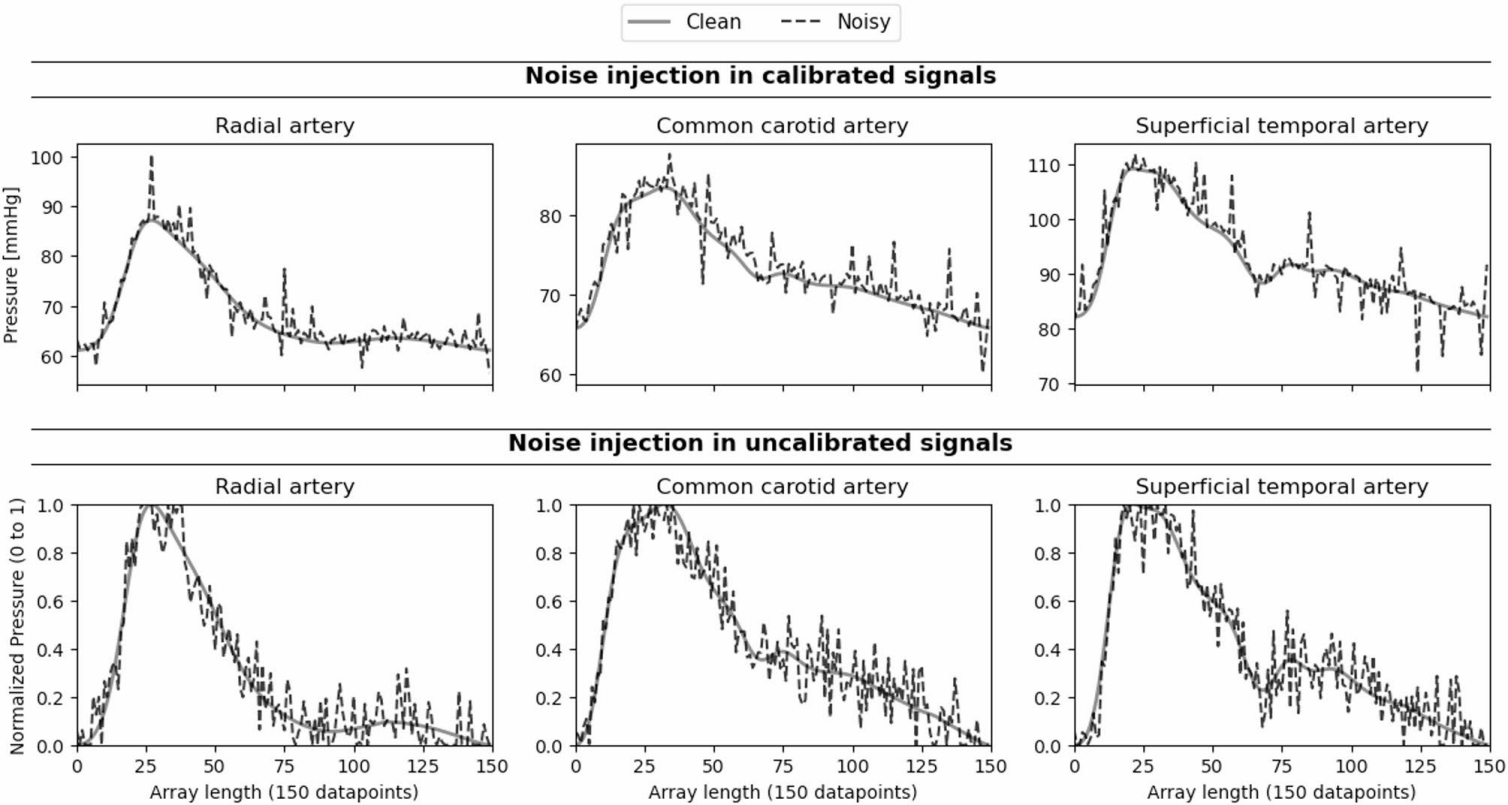
Noise injection examples to pressure signals, calibrated (above) and uncalibrated (below).

The corruption of signals with additive white noise and random spikes is performed after waveform resampling and exemplified in Fig. 4. In the noisy cases, the machine learning models are trained and assessed using the noisy waveforms, keeping model hyperparameters the same as in the respective noise-free cases.

### Statistical analysis

All statistical analyses were performed in python. A *p*-value less than 0.05 was considered statistically significant. Shapiro–Wilk test was used to assess normality and non-parametric Mann–Whitney U test was used for continuous variables (cardiac output and mean aortic pressure) to evaluate differences between the means of the normodynamic and hyperdynamic groups.

Linear regression analysis on the estimated and reference data yielded the slope and intercept of the regression line as well as the *p*-value for the hypothesis test of the slope. Bland–Altman analysis was used to obtain the bias and limits of agreement (LoA) corresponding to a 95% confidence interval.

Intrasite differences between calibrated and normalized Pearson’s r values were assessed using paired t-test following Fischer’s z-transformation of r values.

Intersite differences were compared with the scale-independent coefficient of variation, calculated as the standard deviation divided by the mean, expressing the relative variability of performance metrics between sites as a percentage.

To assess model performance, we used the following standard regression metrics. Mean absolute error (MAE) was calculated as the average of the absolute differences between predicted and reference (ground truth) values. The Normalized Root Mean Square Error (nRMSE) was based on the RMSE, defined as the square root of the average of squared difference between predictions and reference values. The nRMSE expresses the RMSE as a percentage of the range of the target variable (here, CO), allowing for scale-independent interpretation. It is computed as

nRMSE = 100 × RMSE/(y_max_ − y_min_), where y_max_ and y_min_ are the maximum and minimum reference values of CO.

## Results

### Cardiac output estimation from calibrated and uncalibrated pressure traces

**Fig. 5:**
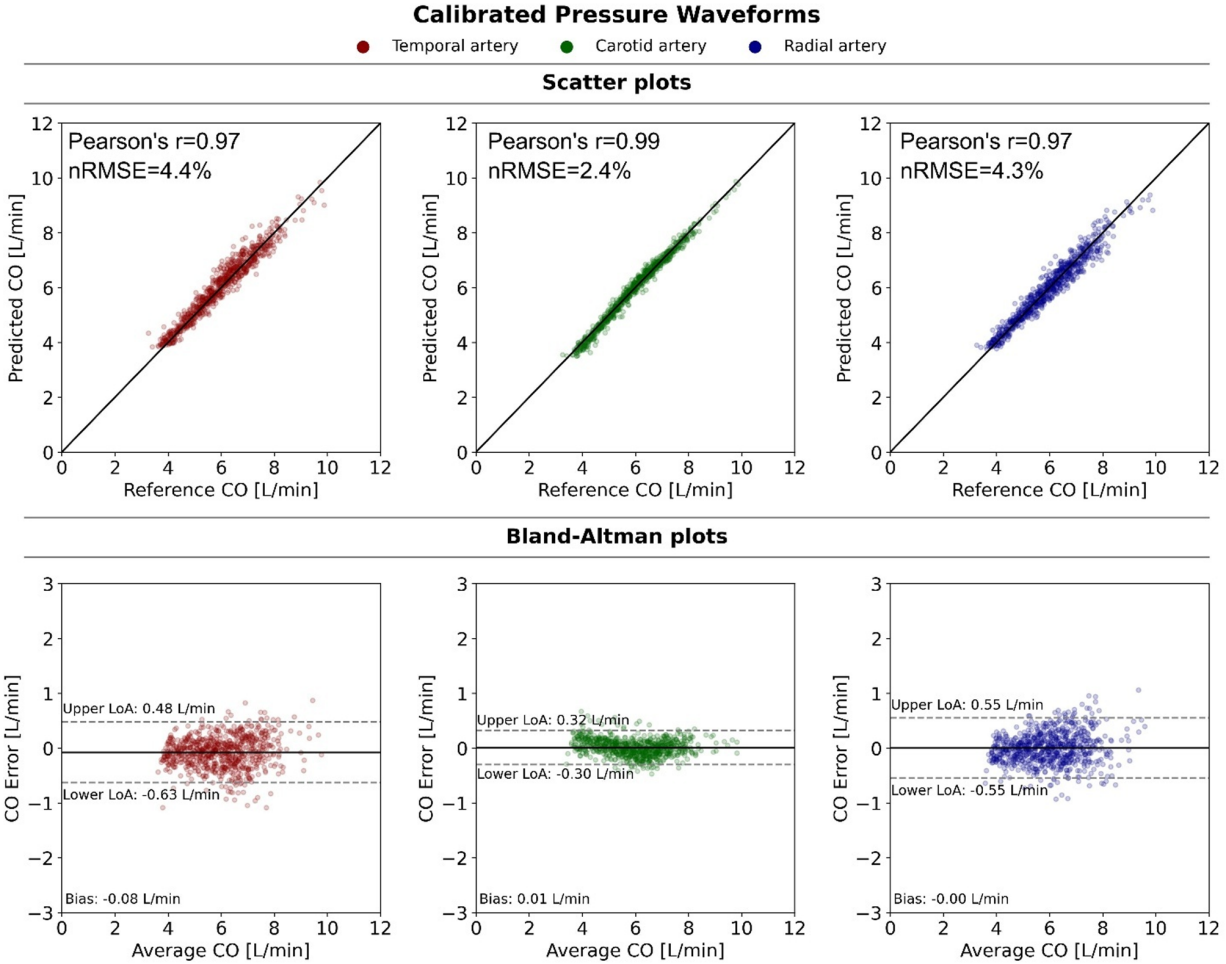
Predicted cardiac output (CO) from calibrated blood pressure waveforms at the temporal, carotid and radial arteries. Top: Scatter plots of CNN-predicted against reference CO values. Bottom: Bland–Altman plots. nRMSE: normalized root mean square error, LoA: limit of agreement.

For each of the CNN models taking as input the calibrated pressure waveforms from the superficial temporal, carotid and radial artery, the predicted cardiac outputs were compared against the ground truth CO values provided by the simulations. To compare the performance of the CNN models consistently, each model was configured with its respective optimal hyperparameters and assessed against the same holdout (test) set of hemodynamic cases, which was excluded from hyperparameter tuning or model training. Scatter plots and Bland–Altman analysis for each arterial location are presented in Fig. 5, demonstrating comparable performance in the predictive ability of CO. The relevant performance metrics are reported in Table 3. CO estimations based on the calibrated carotid artery waveforms result in slightly higher Pearson’s r coefficient and lower nRMSE (r = 0.99 and nRMSE = 2.4%) compared to the calibrated temporal (r = 0.97, nRMSE = 4.4%) and radial signals (r = 0.97, nRMSE = 4.3%), yet all model variants yield high correlation coefficients and relatively narrow limits of agreement.

**Table 3:** CO prediction from clean (noise-free) signals: effect of calibration and arterial location on model performance.

Input BP signal	Model	r	nRMSE (%)	MAE (L/min)	Bias [LoA] (L/min)	Slope	Intercept (L/min)
Temporal artery							
Calibrated	CNN_1_	0.97	4.4	0.23	− 0.08 [− 0.63, 0.48]	0.95	0.39
Uncalibrated	CNN_2_	0.94	6.9	0.36	− 0.18 [− 1.00, 0.65]	0.84	1.10
Carotid artery							
Calibrated	CNN_3_	0.99	2.4	0.12	0.01 [− 0.30, 0.32]	1.02	− 0.11
Uncalibrated	CNN_4_	0.95	6.1	0.29	− 0.11 [− 0.88, 0.65]	0.92	0.60
Radial artery							
Calibrated	CNN_5_	0.97	4.3	0.22	0.00 [− 0.55, 0.55]	0.94	0.38
Uncalibrated	CNN_6_	0.93	7.8	0.41	− 0.24 [0.66, − 1.13]	0.88	0.93

*BP* blood pressure, *r* Pearson’s correlation coefficient, *nRMSE* normalized root mean square error, *MAE* mean absolute error, *LoA* limits of agreement. For all slopes, p < 0.001. Nonsignificant difference between calibrated-r and normalized-r values (p = 0.07, paired t-test on z-transformed r values).

**Fig. 6:**
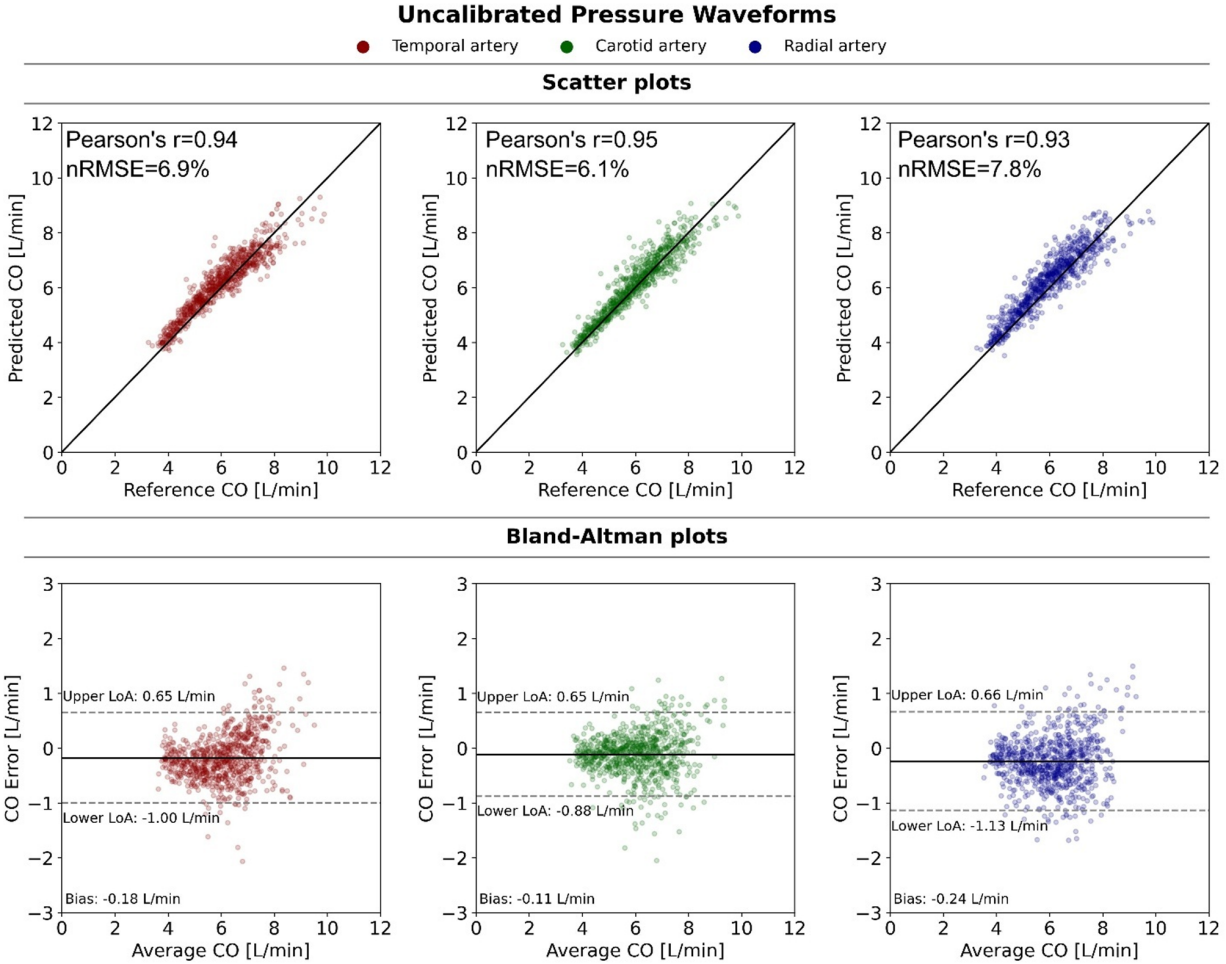
Predicted cardiac output (CO) from uncalibrated blood pressure waveforms at the temporal, carotid and radial arteries. Top: Scatter plots of CNN-predicted against reference CO values. Bottom: Bland–Altman plots. nRMSE: normalized root mean square error, LoA: limit of agreement.

To evaluate the impact of calibration, we additionally assessed CNN models fed with amplitude-normalized pressure waveforms (unitless pressure, varying from 0 to 1) recorded at the same arterial locations. The performance of models based on uncalibrated signals is summarized in Table 3 and illustrated in Fig. 6. Interestingly, models with uncalibrated input signals still perform at satisfactory levels (r ≥ 0.93, MAE ≤ 0.41 L/min), albeit with wider limits of agreement with respect to their calibrated counterparts and modestly lower r values (by no more than 4%). Intersite differences across arterial locations were minimal (coefficient of variation in r-values of calibrated and uncalibrated values 1.2% and 1.1% respectively), presenting a slight advantage in estimations from the common carotid location.

### Effect of noise injection on predictive ability of CNNs

**Fig. 7:**
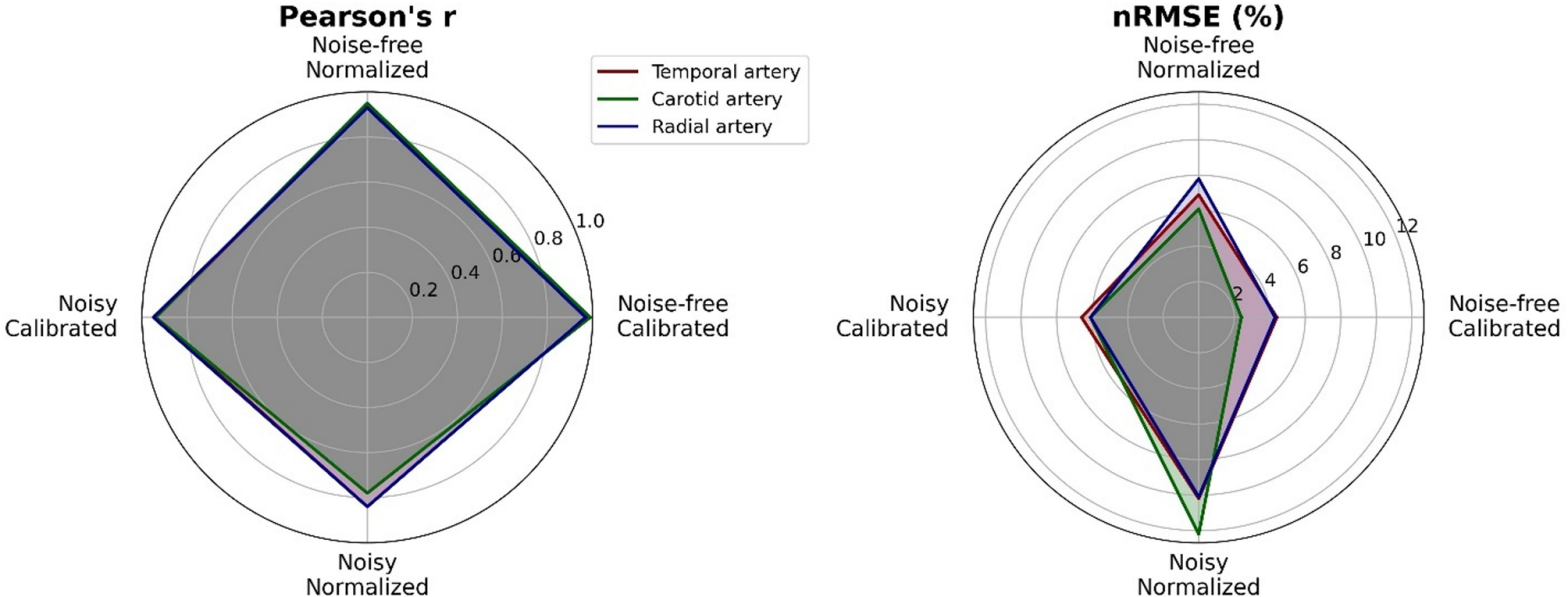
Radar plots comparing model performance across different input signal conditions (calibrated versus normalized/uncalibrated, with and without noise) at three arterial pressure measurement locations.

To emulate real-world blood pressure measurement artefacts, input signals were distorted by additive noise and random spikes. Performance metrics of models based on noisy signals are presented in Table 4. As anticipated, distortion of the input signal degrades the performance of all models, with significantly smaller Pearson’s r correlation coefficient values in noisy versus noise-free models (*p* < 0.01, paired t-test on z-transformed r values pooled across locations and calibration status). In contrast to the noise-free models where calibration doesn’t affect performance, in noisy models it marks a significant impact: Pearson’s r coefficients drop significantly between noisy calibrated and noisy uncalibrated models (*p* < 0.01, paired t-test on z-transformed r values pooled across locations). Scatter and Bland–Altman plots for the noisy input signal models can be found in the Supplementary Material (Figs. S2 and S3). Intersite variability in r values between arterial locations is low (coefficient of variation 0.5% and 4% for noisy calibrated and noisy uncalibrated signals respectively). The impact of both calibration and noise on model performance across all three arterial locations is illustrated in Fig. 7. The radar plots highlight the relative consistency in performance degradation under different calibration and noise conditions, providing a comparative overview of model performance at each site.

**Table 4:** CO prediction from noisy signals: effect of calibration and arterial location on model performance.

Noisy input BP	Model	r	nRMSE (%)	MAE (L/min)	Bias [LoA] (L/min)	Slope	Intercept (L/min)
Temporal artery							
Calibrated	CNN_1, noise_	0.94	6.6	0.44	0.15 [0.65, − 0.95]	0.86	0.71
Uncalibrated	CNN_2, noise_	0.84	10.2	0.67	0.04 [− 1.28, 1.36]	0.79	1.21
Carotid artery							
Calibrated	CNN_3, noise_	0.94	6.1	0.41	− 0.02 [− 0.82, 0.77]	0.94	0.41
Uncalibrated	CNN_4, noise_	0.78	12.2	0.80	− 0.20 [− 1.73, 1.33]	0.75	1.72
Radial artery							
Calibrated	CNN_5, noise_	0.95	6.1	0.41	0.16 [− 0.58, 0.89]	0.89	0.51
Uncalibrated	CNN_6, noise_	0.84	10.1	0.67	0.16 [− 1.12, 1.43]	0.72	1.50

*BP* blood pressure, *r* Pearson’s correlation coefficient, *nRMSE* normalized root mean square error, *MAE* mean absolute error, *LoA* limits of agreement. For all slopes, *p* < 0.001.

## Discussion

In absence of an undisputed noninvasive method to measure and monitor cardiac output, we develop an AI-based methodology to extract cardiac output from the intrinsic shape of pressure waves, independent of pressure calibration amplitude or sampling frequency. We investigate whether the recording site, calibration state or noise distortion of the pressure signal impact the predictive ability of the model. We observe that pressure traces (both calibrated and uncalibrated) from the superficial temporal, common carotid or radial artery, all easily measurable with tonometers, yield cardiac output values with comparable accuracy.

Rather than extracting informative features from the raw waveforms using pulse wave or pulse contour analysis, inevitably informed by prior knowledge on the systemic circulation, the CNN model proposed herein processes input pulse waves without regard to the underlying cardiovascular physiology. The hierarchical feature extraction performed by the CNN is therefore physiology-agnostic and unbiased. Recently, a similar approach was proposed for AI-based estimation of vascular age based on peripheral pressure waveforms from the carotid, brachial or radial arteries^15^.

We observe that CNN models with input waveforms recorded at different arterial locations perform similarly in predicting cardiac output. Intersite differences between arterial locations are minimal; differences in achieved levels of Pearson’s r correlation coefficients do not exceed 4%. Nonetheless, a slight performance advantage was seen when using waveforms from the common carotid artery. While modest, this trend is physiologically plausible: carotid artery pressure is often used as a surrogate of central pressure owing to the proximity of the two arterial sites^27,28^, and the carotid artery’s properties relate to cardiac function^29^.

Despite minimal variations, our overall findings suggest that the wealth of information encoded in peripheral arterial pressure waveforms is preserved within the arterial tree, notwithstanding the particular recording site’s proximity to the heart, or the complexity of the arterial route leading from the heart up to the specific measurement site. The propagating arterial pulse is subject to shape alterations (wave reflections, central-to-peripheral pulse pressure amplification), owing to local vessel properties and, at the same time, to the rest of the arterial network. While information in its original form (wave morphology) is altered, no functional or physiological “message” carried by the pulse wave is lost as we move towards peripheral sites. Candidate peripheral pressure measurement locations appear essentially interchangeable and, ultimately, selection of the recording site serving as pulse wave input can be guided solely by local measurement accuracy and ease.

Remarkably, the intrinsic peripheral pressure wave morphology (independent of calibrated pressure amplitude) suffices to predict CO with similar accuracy in all arterial locations. Indeed, this finding aligns with a prior study (on the same in silico dataset) on reasonably accurate CO estimation from uncalibrated radial pressure waveforms using a feedforward neural network^17^. As observed, noise injection affected model performance quite consistently across all locations, with similar trends in Pearson’s correlation and nRMSE levels observed for each site.

The idea of deriving cardiac output from arterial blood pressure measurements is certainly not novel; such endeavors form the basis of invasive, minimally invasive and noninvasive cardiac output estimation techniques using pulse wave analysis or the pulse contour method^30,31^. Notably, within the gamut of noninvasive continuous CO monitoring techniques certain commercial systems rely on the volume clamp method for continuous blood pressure measurement (using a finger cuff) and subsequently estimate CO by fitting a three-element Windkessel model (ClearSight ex-Nexfin system, Edwards Lifesciences) or by means of pulse wave analysis of the acquired waveform (CNAP, CNSystems Medizintechnik, Austria)^32^. Arguably the most relevant to our approach among existing commercial systems is the one employing automated radial artery applanation tonometry (for continuous blood pressure measurement) and a proprietary algorithm for stroke volume estimation requiring no external calibration (T-Line, ex Tensys medical, HBM Healthcare; DMP-Life, Daeyomedi Co., South Korea)^6,33,34^. Such Windkessel or pulse wave analysis frameworks however make a priori assumptions to engineer information extraction from the pulse waveform.

Data-driven, rather than physiology-based, approaches to extract cardiac output from arterial waveforms were first employed more than 30 years ago^35^ and have recently attracted renewed attention^36–41^. It can be envisaged that in the future such AI-based algorithms may assist (or eventually replace) proprietary pulse wave analysis-based algorithms in bespoke CO monitoring devices. The current study proposes a deep learning method to estimate CO, but, more so, pursues the fundamental question of whether the location of arterial pressure measurement matters for CO determination, and what, if any, is the location-specific effect of calibration and noise addition. Our results suggest an equivalence of arterial sites, yet further studies on the interpretability of the AI algorithms are warranted to determine whether signals originating from different locations are processed in a comparable way and thereby ensure fairness in the comparison of locations. Furthermore, given that signal quality in clinical practice spans a continuum from clean to noisy, future work may explore hybrid models trained on both clean and noisy waveforms to enhance model robustness.

The current work builds upon a previous in silico study which proposed a simple Feedforward Neural Network (FNN) with one fully connected hidden layer containing 32 neurons to predict CO from pressure traces obtained at the radial artery, either calibrated or uncalibrated (amplitude-normalized from 0 to 1), fed to the FNN as vectors of 151 points^17^. The FNN treated each input feature of the 151-point input pressure arrays independently, assuming no inherent relationship or order between them. In the current framework we opted for a 2-layered CNN architecture for the required time-series regression task, which leverages convolutional operations to capture local dependencies or sequential structures present in the time-series data. Unlike FNNs, which treat each data point independently, CNNs are better suited for waveform data because they can detect patterns and relationships between neighboring points—much like how they are used in image processing to recognize shapes or edges. This ability to capture temporal structure makes CNNs more appropriate for modeling pressure waveforms, where the order and local trends of data points carry important physiological meaning. Another difference in our approach pertains to the preprocessing steps to address the non-uniformity of heart cycle durations among the in silico cases, and hence the non-uniformity of their pressure waveform lengths. In Bikia et al.^17^ all signals were resampled to 128Hz and a uniform pressure array length of 151 points was achieved through padding with a trail of dummy values at the end of each case’s pressure signal. Given that trailing dummy values during model training may interfere with the training process, we opted to up- and down-sample all signals to reach uniform input signal length of 150 points.

While our study provides important insights, several limitations should be acknowledged. In our pipeline, noise was injected after resampling the waveforms to a fixed length. While this ensured consistency across samples, it does not fully replicate clinical preprocessing workflows, where noise is typically present in raw signals and addressed before resampling. In future work we plan to adopt a more realistic sequence—noise injection followed by filtering and then resampling—to better mirror clinical signal processing. Another limitation of the current approach is that waveform resampling to a fixed length removes explicit timing information, including heart rate, which is an essential determinant of cardiac output. While this strategy was effective in our synthetic dataset, the loss of timing-related features including heart rate may limit transferability beyond synthetic data. Future work will explore the inclusion of explicit timing features or sequence-aware architectures to address this limitation and improve generalizability to clinical data. Additionally, applying a fixed-size convolutional kernel to resampled waveforms (hence of varying timestep duration) may introduce inconsistencies, since there is no longer a consistent temporal scale across signals. In real-world settings, such temporal inconsistencies could negatively affect model performance.

The data of the current study are drawn from an in silico dataset previously generated using a validated 1-D model of the systemic arterial circulation. These idealized in silico-generated pressure recordings are undoubtedly far from clinical reality, while the underlying distributions of the synthetic data merit careful consideration, as shown in this study. The hyperdynamic states simulated in the dataset are less common clinically and often not as urgent as hypodynamic conditions. Importantly, the synthetic population does not include severe hypotensive or hypodynamic scenarios, which we acknowledge limits generalizability. It should also be emphasized that the apparent success of design choices and methods in synthetic data does not guarantee their robustness in clinical applications, where variability and noise are substantially greater. Yet such virtual datasets are instrumental in proof-of-concept, methodology development and refinement studies, where clinical data is hard to obtain or scarce. Interpreting results from in silico-based studies should be exercised with caution, nevertheless such studies constitute valuable steps forward towards achieving efficient noninvasive hemodynamic monitoring and improving patient outcomes.

## Electronic supplementary material

Below is the link to the electronic supplementary material.

Supplementary Material 1

## Data Availability

The data that support the findings of this study are available from the corresponding author upon reasonable request. Correspondence and requests for materials should be addressed to Lydia Aslanidou.
