# Supplementary material for: Machine learning-enabled estimation of cardiac output from peripheral waveforms is independent of blood pressure measurement location in an in silico population
Source: Sci Rep. 2025 Jul 15;15:25562. doi: 10.1038/s41598-025-10492-2 (PMC12264289; doi:10.1038/s41598-025-10492-2)
Supplement: Supplementary file 1 — Supplementary Material 1 [file 41598_2025_10492_MOESM1_ESM.pdf]

Machine learning-enabled estimation of cardiac output from peripheral waveforms is independent of blood pressure measurement location in an in silico population

Lydia Aslanidou[1*], Georgios Rovas[1], Ramin Mohammadi[1], Sokratis Anagnostopoulos[1], Cemre Çelikbudak Orhon[1], and Nikolaos Stergiopulos[1]

[1]Laboratory of Hemodynamics and Cardiovascular Technology (LHTC), EPFL, Lausanne, Switzerland

SUPPLEMENTARY MATERIAL

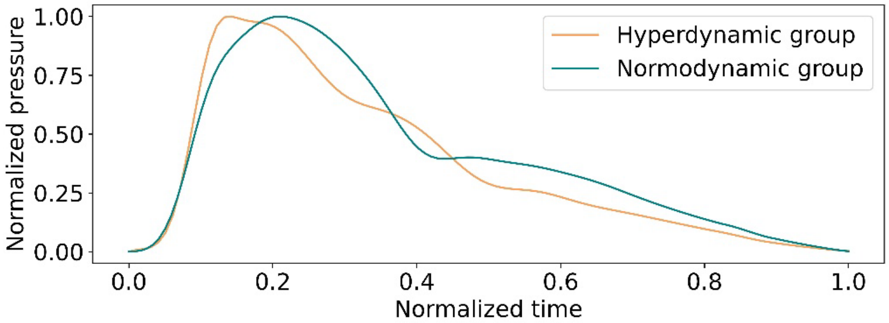

Figure S1. Individual pressure traces from the hyperdynamic and normodynamic groups of the in silico dataset.

Table S1. Hyperparameters obtained from Bayesian optimization for each of the CNNs, based on input waveforms' source location and calibration status. Optimization was performed using the *optuna* package.

Hyperparameter	Temporal artery BP		Carotid artery BP		Radial artery BP	
	Calibrated CNN_1	Normalized CNN_2	Calibrated CNN_3	Normalized CNN_4	Calibrated CNN_5	Normalized CNN_6
Learning rate	$4.4 \cdot 10^{-4}$	$2.1 \cdot 10^{-4}$	$1.8 \cdot 10^{-4}$	$1.0 \cdot 10^{-4}$	$6.2 \cdot 10^{-5}$	$3.2 \cdot 10^{-4}$
Batch size	8	8	8	16	8	8
Filters 1	32	64	16	16	32	16
Filters 2	128	128	256	128	256	64
Kernel size	9	9	7	5	9	5
Dense units	128	128	256	128	64	64
Dropout rate	0.03	0.21	0.49	0.14	0.31	0.32
Epochs	37	42	51	48	47	32

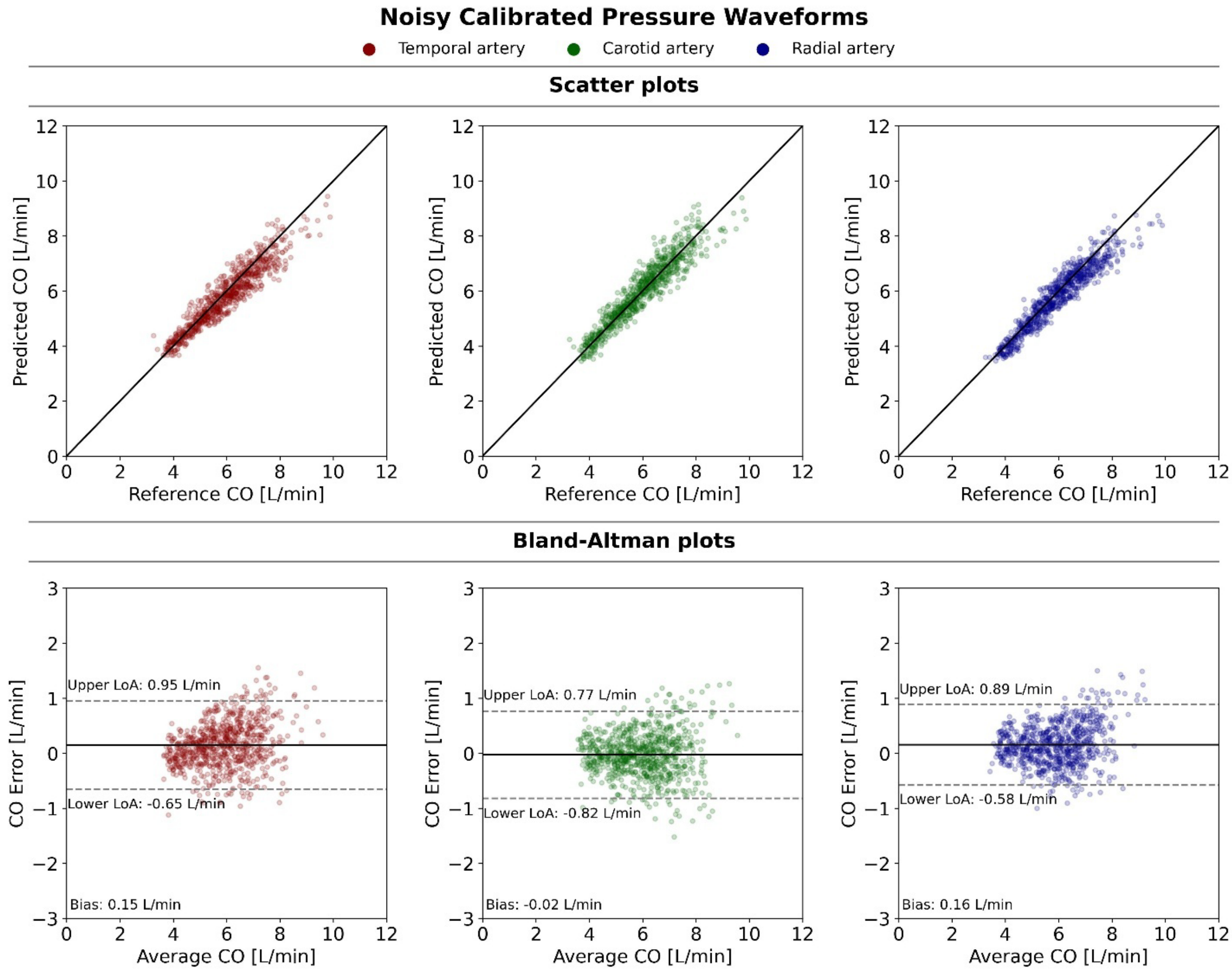

Figure S2. Performance of CNNs with noisy calibrated input signals.

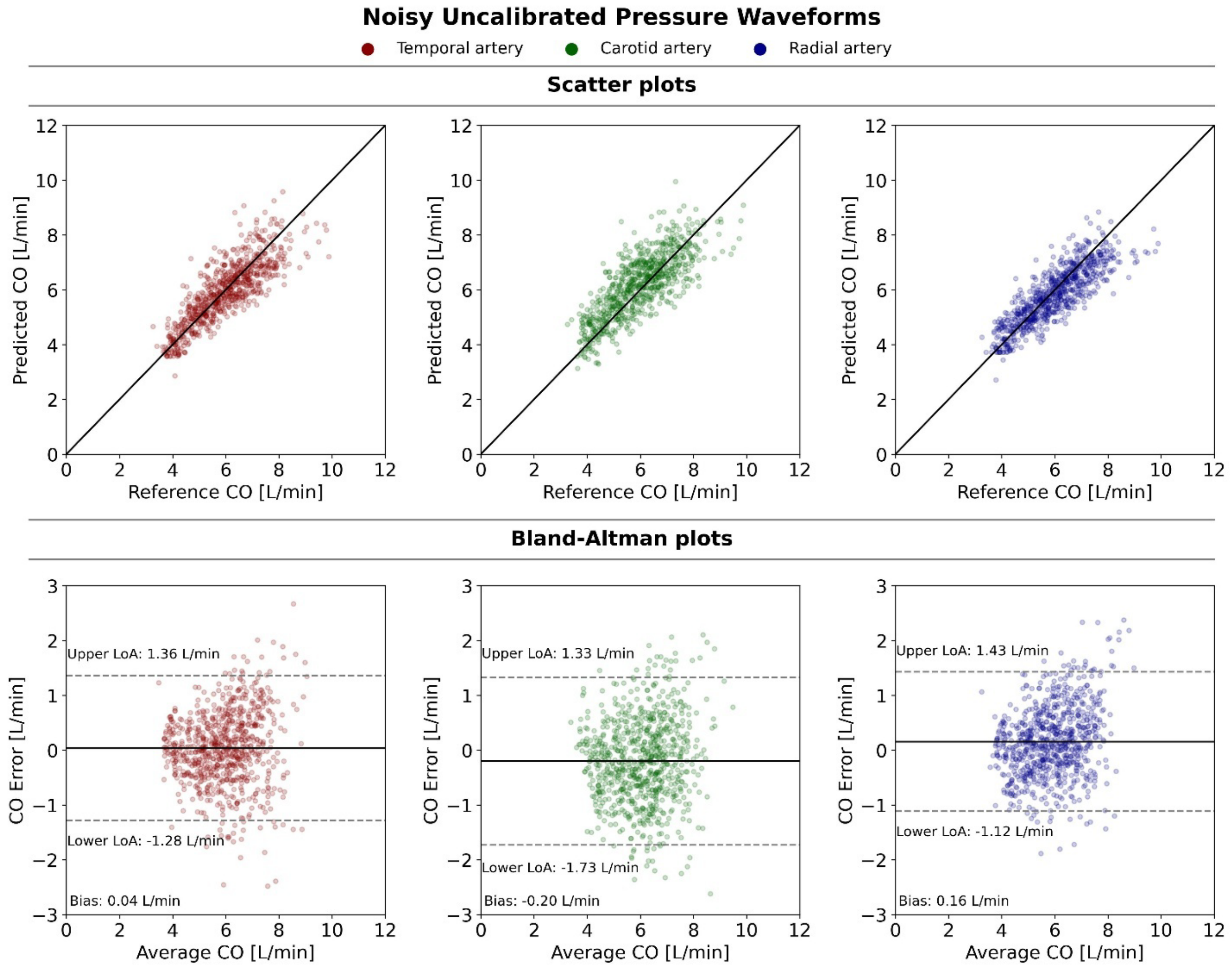

Figure S3. Performance of CNNs with noisy calibrated input signals.
